# Short-term clinicopathological outcome of neoadjuvant chemohormonal therapy comprising complete androgen blockade, followed by treatment with docetaxel and estramustine phosphate before radical prostatectomy in Japanese patients with high-risk localized prostate cancer

**DOI:** 10.1186/1477-7819-10-1

**Published:** 2012-01-04

**Authors:** Shintaro Narita, Norihiko Tsuchiya, Teruaki Kumazawa, Shinya Maita, Kazuyuki Numakura, Takashi Obara, Hiroshi Tsuruta, Mitsuru Saito, Takamitsu Inoue, Yohei Horikawa, Shigeru Satoh, Hiroshi Nanjyo, Tomonori Habuchi

**Affiliations:** 1 Department of Urology, Akita University School of Medicine, Akita, Japan; 2 Department of Urology, Senboku General Hospital, Daisen, Japan; 3 Department of Pathology, Akita University School of Medicine, Akita, Japan

**Keywords:** chemohormonal therapy, prostate cancer, docetaxel, estramustine phosphate, complete androgen blockade, androgen deprivation

## Abstract

**Background:**

To assess the outcome of neoadjuvant chemohormonal therapy comprising complete androgen blockade followed by treatment with docetaxel and estramustine phosphate before radical prostatectomy in Japanese patients with a high risk of localized prostate cancer (PCa).

**Methods:**

Complete androgen blockade followed by 6 cycles of docetaxel (30 mg/m^2^) with estramustine phosphate (560 mg) were given to 18 PCa patients before radical prostatectomy. Subsequently, the clinical and pathological outcomes were analyzed.

**Results:**

No patients had severe adverse events during chemohormonal therapy, and hence they were treated with radical prostatectomy. Two patients (11.1%) achieved pathological complete response. Surgical margins were negative in all patients. At a median follow-up of 18 months, 14 patients (77.8%) were disease-free without PSA recurrence. All 4 patients with PSA recurrence had pathologic T3b or T4 disease and 3 of these 4 patients had pathologic N1 disease.

**Conclusion:**

We found that neoadjuvant chemohormonal therapy with complete androgen blockade followed by treatment with docetaxel and estramustine phosphate before radical prostatectomy was safe, feasible, and associated with favorable pathological outcomes in patients with a high risk of localized PCa.

## Background

Prostate cancer (PCa) is currently the most common malignancy in men, and the second leading cause of cancer-related death in Western populations [1]. In Japan, morbidity and mortality due to PCa have been increasing. This increase may be due to changes in dietary habits, increased screening for PCa, and an expanding elderly population. Due to recent advances in surgical procedures, the operative outcomes have been reported to improve [2]. However, men with high-risk localized PCa [as defined by the parameters including serum prostate-specific antigen (PSA) levels, clinical stage, and histological grade] have a significantly higher possibility of biochemical relapse than the control groups [3,4]. To improve the outcome of local therapy, several groups conducted neoadjuvant hormonal therapy before radical prostatectomy. However, a recent systematic review and meta-analysis showed that neoadjuvant hormone therapy before prostatectomy does not improve the overall survival and disease-free survival, while it does improve the pathological outcome [5].

Docetaxel remains the standard first-line chemotherapy in castration resistant PCa based on two large randomized trials demonstrating improved overall survival compared to mitoxantrone [6,7]. However, in the setting of neoadjuvant therapy, chemotherapy with docetaxel as a single agent has minimal value in PCa patients treated with radical prostatectomy without pathological CR [8,9]. Recent studies showed the benefit and clinicopathological results of neoadjuvant chemohormonal therapy in patients with high-risk localized PCa, including the combination chemohormonal therapy with other cytotoxic drugs that includes estramustine phosphate [10-13]. Although the true effect remains controversial because of the large variations in the schedule of neoadjuvant chemohormonal therapy and lack of results from large randomized trials, a neoadjuvant chemohormonal therapy could have a role in the treatment of patients with a high risk of PCa.

Here, we conducted a small pilot study to assess the short-term outcome of neoadjuvant chemohormonal therapy comprising complete androgen blockade followed by treatment with docetaxel and estramustine phosphate after radical prostatectomy in Japanese patients with high-risk localized PCa.

## Methods

### Patient selection

Eligibility criteria included patients with histopathologically documented, locally advanced "high risk" of PCa who were judged to be candidates for radical prostatectomy performed at Akita University Hospital, Akita, Japan. "High-risk" disease was defined as any of the following requirements such as clinical stage T3, preoperative PSA level of 15 ng/mL or greater, and/or Gleason score greater than 9. The definition of the criteria for high-risk prostate cancer was based on our previous data of radical prostatectomies without neoadjuvant or adjuvant hormonal therapy. In the previous data, a more significant difference in the PSA recurrence-free survival rate was observed when a Gleason score of 9 or 10 was defined as high-risk criteria than when a score of 8-10 was included. Regarding the PSA level, we also found that a more significant difference in the PSA recurrence-free survival rate was observed when the cut-off value was 15 ng/mL than when the value was 20 ng/mL. Staging was according to the TNM classification (UICC 1997). Clinical stage was assigned on the basis of digital rectal examination findings, CT imaging, and bone scintigraphy. Patients were excluded from participating in the study if they received prior therapy for PCa, had prior invasive malignancy, or any serious comorbidities. Patients having ECOG performance status with 2 or greater were excluded. All patients provided written informed consent. The study was approved by the Institutional Review Boards (Ethical Committee) of Akita University.

### Treatment protocol

Treatment consisted of complete androgen blockade with subcutaneous administration of 11.25 mg leuprorelin once every 3 months and oral administration of 81 mg bicalutamide for the first 12 weeks. Next, docetaxel was administered intravenously at a dose of 30 mg/m^2 ^weekly for 6 consecutive weeks along with oral administration of 560 mg estramustine phosphate twice a day. Estramustine phosphate was allowed to be decreased to 50% if patients had dysphagia, nausea or vomiting. Patients were withdrawn from the study if hematologic, hepatic or other grade 3 or grade 4 non-hematologic toxicities did not resolve within 3 weeks.

Patients then underwent modified extended radical prostatectomy as reported by Miyake et al. [14], and a bilateral pelvic lymphadenectomy according to a standardized technique [15] under general anesthesia within 4 weeks after the last administration of docetaxel. One patient who was suspected of having direct invasion into the bladder underwent radical cystoprostatectomy after neoadjuvant chemohormonal therapy.

### Monitoring

After radical prostatectomy, patients were followed up every 3 months and assessed for serum PSA levels. Biochemical disease progression was defined as the serum PSA level greater than 0.2 ng/mL.

### Pathologic evaluation

Standard pathological examination of prostatectomy specimens was performed at Akita University Hospital. The pathological response to the neoadjuvant therapy was assessed by a single pathologist (H.N.). Pathological complete response was defined as complete eradication of the tumor. According to the General rule for Clinical and Pathological Studies on PCa recommended by the Japanese Urological Association, Japanese Society of Pathology, and the Japan Radiological Society [16], pathological changes after chemohormonal therapy were graded as follows: grade 0a (viable cells in all cancer areas and no degenerated cells), grade 0b (viable cells in all the specimen with degenerated cells), grade 1 (viable cells in half or less of cancer areas), grade 2 (viable cells in more than half of cancer areas), grade 3a (non-viable cancer cells), and grade 3b (no cancer cells). No detectable viable cell (grade 3) in whole specimens was defined as pathological complete response (CR).

### Endopoints and Statistical analysis

The primary end point was the complete pathological CR. Additional endpoints were safety, feasibility, and time to biochemical failure. Toxicity was graded using Common Toxicity Criteria (version 4.0). PSA progression-free survival was defined as the time of surgery to the date of first PSA recurrence and was calculated using the Kaplan-Meier method.

## Results

### Patient characteristics

Eighteen patients were enrolled in this study from March 2006 to December 2010. The pretreatment characteristics of the study population are listed in Table 1. The median age was 67 years (range, 57-69 years), and the median pretreatment serum PSA level was 25.8 ng/mL (range, 5.1-45.1 ng/mL). The ECOG performance status of all patients was 0. The pretreatment Gleason score was 5 in 1 patient (6.3%), 6 in 1 patient (6.3%),7 in 3 patients (18.8%), 8 in 1 patient (6.3%), 9 in 7 patients (43.8%), and 10 in 3 patients (18.8). Nine patients (56.3%) had clinically organ-confined disease, and 6 patients (37.5%) had cT3 disease. Six patients (33.3%) had one of the 3 risk factors in our high-risk criteria, 7 patients (38.9%) had 2 risk factors, and 5 patients (27.8%) had all 3 risk factors.

**Table 1:** Patient characteristics

		No. patients	%
Total no. of patients		18	
Age, years			
Median (range)		67 (57-69)	
ECOG PS			
	0	18	100
Serum PSA, ng/mL			
Median (range)		25.8 (5.1-45.1)	
Clinical stage, T			
	1	3	18.8
	2	6	37.5
	3	6	37.5
	4	1	6.3
Gleason score			
	5	1	6.3
	6	1	6.3
	7	3	18.8
	8	1	6.3
	9	7	43.8
	10	3	18.8

### Adverse events

The prevalence of adverse events are summarized in Table 2. Grade 3 or grade 4 hematological adverse events were not observed. The elevation of serum transaminase level with grade 3 was found in 1 patient, who recovered within 4 weeks. In patient number 3 and 14, docetaxel administration was abandoned after 5 courses. In patient number 12, only 3 courses of docetaxel administration was given because of mild nausea (Table 3). With respect to postoperative complications, 1 patient with a ureteral injury required cystoureterostomy 15 days after radical prostatectomy. Another patient who had a lymphocele required drainage for 25 days.

**Table 2:** Adverse events

	Grade			
	
	I	II	III	IV
Hematological toxicity				
Leukopenia	1 (6.3%)	0	0	0
Anemia	2 (11.1%)	1 (6.3%)	0	0
Thrombocytopenia	0	0	0	0
Non-hematological toxicity				
Nausea/Vomiting	2 (11.1%)	2 (11.1%)	0	0
Diarrea	1 (6.3%)	0	0	0
Dysgeusia	1 (6.3%)	0	0	0
transamirase increased	0	1 (6.3%)	1 (6.3%)	0

**Table 3:** Summary of patient outcomes

**No**.	Age (yrs)	BaselinePSA (ng/ml)	Gleason score	Clinical stage	Preoperative PSA (ng/ml)	Pathological stage	Surgical margin	Pathological change	PSA progression (mo)
1	58	17.0	7	T2aN0	0.083	T2bN0	negative	2	No
2	68	21.5	7	T1cN0	0.078	T2bN0	negative	2	No
3	69	15.0	9	T2aN0	0.045	T2aN0	negative	2	No
4	69	23.4	6	T3aN0	0.143	T2bN0	negative	2	No
5	67	42.9	9	T3aN0	0.133	T2bN0	negative	2	No
6	67	5.1	10	T1bN0	0.002	T0N0	negative	3b	No
7	57	35.9	9	T3aN1	0.056	T3bN1	negative	1	6
8	64	28.3	9	T3aN0	0.084	T2bN0	negative	2	No
9	58	45.1	8	T3aN1	0.071	T3bN1	negative	2	No
10	66	37.8	7	T3bN0	0.11	T3bN1	negative	1	6
11	58	61.2	10	T4N0	0.52	T4N1	negative	1	7
12	62	30.7	5	T1cN0	0.498	T2aN0	negative	2	No
13	67	77.0	10	T2bN0	0.335	T3bN0	negative	1	6
14	67	21.4	9	T2bN0	0.132	T2bN0	negative	2	No
15	63	5.4	9	T2aN0	0.132	T2aN0	negative	1	No
16	68	17.3	9	T2cN0	0.036	T0N0	negative	3b	No
17	71	25.3	7	T1cN0	0.017	T2aN0	negative	2	No
18	67	79.8	9	T1cN0	0.932	T2bN0	negative	0b	No

### Surgical outcome

All patients underwent radical prostatectomy and bilateral pelvic lymphadenectomy. The median serum PSA level before surgery was 0.097 ng/mL (range, 0.002-0.932). The tumor characteristics of surgical specimens are listed in Table 3. The pathological changes in our series were grade 0 in 1 patient (0.6%), grade 1 in 5 patients (27.8%), and grade 2 in 10 patients (55.6%). Two patients (11.1%) had no detectable tumor (pathological CR). Surgical margins were negative in all patients. Eleven patients (61.1%) had pathological T2 disease, and 2 patients (11.1%) had involvement of seminal vesicles (T3b). The tumor disseminated into the pelvic lymph nodes (pN1) in 4 patients (22.2%).

### Short term outcome

**Figure 1:**
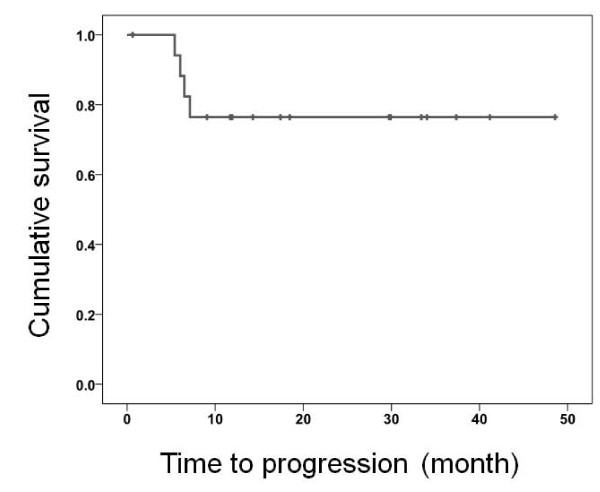
**PSA recurrence-free survival**.

The median follow-up was 18 months (range, 1-49 months). All 4 patients with PSA recurrence (16.7%) had pathological T3b or T4 disease, and 3 of these 4 patients had pathological lymph node positive disease. The Kaplan-Meier curve shows that the PSA recurrence-free survival and median PSA recurrence-free survival was not attained (Figure 1).

## Discussion

In this study, we elucidated the efficacy of the combination of neoadjuvant chemohormonal therapy comprising complete androgen blockade followed by treatment with docetaxel and estramustine phosphate after radical prostatectomy in patients with high-risk of localized PCa. This regimen had acceptable toxicity, negative surgical margins, and two pathological CR.

To our knowledge, only 2 studies have reported the outcome of neoadjuvant chemohormonal therapy using a combination of androgen deprivation followed by treatment with docetaxel and estramustine phosphate [12]. Sella et al. reported that combination therapy with complete androgen blockage, 4 cycles of docetaxel (70 mg/m^2^) on day 2, and estramustine phosphate (280 mg, 3 times daily) from day 1 to day 5 every 21 days in 22 poor prognosis localized PCa patients. They concluded that there was no pathological CR with 63.6% of organ-confined disease, 72.7% of specimen-confined disease, 40.9% of seminal vesicle invasion, and 18.1% of the lymph node dissemination of tumors. At a median follow-up of 23.6 months, 10 patients (45.5%) relapsed. Prayer-Galetti et al. conducted a phase II study with combination chemohormonal therapy with a longer follow-up. Patients in their study received LHRH analog until the PSA nadir, which was followed by a combined regimen of estramustine phosphate (600 mg) and docetaxel (70 mg/m^2^) for four 3-week cycles. Of 22 patients enrolled in their study, 1 patient (5%) achieved pathological CR, and 6 patients (32%) had less than 10% residual tumor. At a median follow-up of 53 months, 14 patients (58%) relapsed with 23 months of median time to recurrence. They also suggested that seminal vesicle invasion and the volume of residual tumor were the predictors of recurrence-free survival. It is difficult to compare the results among this study including the present study due to the variation in eligibility criteria, quality of surgery, difference in the numbers of patients evaluated, and duration of follow-up. However, recent phase I/II studies of docetaxel with oral estramustine have demonstrated comparable efficacy with less toxicity in patients with castration-resistant PCa [17]. The combination of docetaxel and estramustine phosphate is a reliable option in the setting of neoadjuvant therapy. Randomized studies are needed to clarify the role of neoadjuvant chemohormonal therapy.

Within the follow-up period, the present study had 4 patients with PSA recurrence. In addition, all 4 patients with PSA recurrence had local invasive disease (pT3b or pT4). Prayer-Galetti et al. reported that seminal vesicle invasion is an independent prognostic factor for patients with high risk of PCa treated with chemohormonal therapy [10]. Prediction of local invasion may therefore be important for the selection of patients treated with neoadjuvant chemohormonal therapy. Two patients had small enlarged lymph nodes that were detected upon abdominal CT scan before treatment, and thus they were considered to be eligible for neoadjuvant therapy, because of the possibility to have a non-cancerous enlargement of lymph nodes. Considering the pathological results retrospectively, it was found that the enlarged lymph nodes were the lymph node metastases of PCa. However, 1 of these patients had no PSA progression for 18 months. It was also found that patients with pathologically positive lymph nodes had a higher recurrence rate, and thus the prognosis of patients with pathological lymph node dissemination is unclear.

Previous animal studies have shown that simultaneous chemohormonal therapy is better than sequential chemohormonal therapy in reducing disease progression [18]. Clinically, it remains controversial because the timing of combination has differed in each study. However, recent evidence suggests that castrated patients with PCa have a high clearance of docetaxel and they have less adverse effects [19]. In addition, patients in the present study had no severe adverse effect during chemohormonal therapy except the elevation of serum transaminase. Collectively, sequential therapy may be more feasible to neoadjuvant therapy in PCa patients treated with androgen deprivation followed by chemotherapy.

Three patients had unsuccessful completion of the administration of 6 sequential cycles of docetaxel. The reasons for cancellation of the treatment were: (1) difficulty in attending a hospital (2 patients) and (2) grade 2 nausea (1 patient). It would be necessary to validate whether incomplete administration of docetaxel affects the outcome in patients treated with our protocol.

The present study had certain limitations. Firstly, assessing the contribution of each agent for the effect and outcome in patients was difficult, because we could not define if the effect was only due to chemotherapy or due to a late effect of prolonging complete androgen blockade. Secondly, testosterone was suppressed by the administration of estramustine phosphate, which has a long half-life. In the light of this fact, the effect of estramustine phosphate should be considered to assess PSA recurrence after surgery. The serum testosterone levels need to be continuously assessed after surgery. Finally, the duration of follow-up was only 18 months, which was shorter than that in previous reports. Prayer-Galleti et al. showed that the median time to PSA recurrence in patients treated with combination therapy was 23 months [10]. Further validation is required to assess the long-term outcome and suitable patients for neoadjuvant chemohormonal therapy with a high risk of localized PCa.

## Conclusions

We found that neoadjuvant chemohormonal therapy with complete androgen blockade followed by treatment with docetaxel and estramustine phosphate before radical prostatectomy was safe, feasible, and associated with favorable pathological outcomes in patients with a high risk of localized PCa. Although ongoing randomized phase II trials such as CALGB 90203 and GETUG12 may help to assess the efficacy of neoadjuvant chemohormonal therapy, our regimen may be a candidate for the optimal chemohormonal therapy in patients with high-risk localized PCa.

## Competing interests

The authors declare that they have no competing interests.

## Authors' contributions

SN is a main conductor of this study, participated in the design of the study, and drafted the manuscript. NT, TK, SM and KN participated in the acquisition of data. TO, HT, MS, TI, YH, and SS participated in the collection of materials and data of the study. TH designed and organized the study, and wrote the manuscript. HN was in charge of  the histopathological assessment. All authors read and approved the final manuscript.
